# Effects of Swimming-Specific Repeated-Sprint Training in Hypoxia Training in Swimmers

**DOI:** 10.3389/fspor.2020.00100

**Published:** 2020-08-11

**Authors:** Marta Camacho-Cardenosa, Alba Camacho-Cardenosa, Adrián González-Custodio, Víctor Zapata, Guillermo Olcina

**Affiliations:** Faculty of Sport Sciences, University of Extremadura, Cáceres, Spain

**Keywords:** altitude, performance, water sports, repeated-sprint ability, hypoxemia

## Abstract

The aim of this study was to investigate the effect of a 4 weeks in-water swimming-specific repeated-sprint training in hypoxia (RSH) compared to similar training in normoxia (RSN). Following a repeated-measures, counterbalanced cross-over design, 10 swimmers were requested to perform two trials consisting of in-water repeated sprints in hypoxic (RSH, simulated 4,040 m; FiO_2_ = 13.7%) or normoxic (RSN, 459 m, FiO_2_ calibrated = 20.9%) conditions. In both conditions, 8 additional exercise including 3 sets of 5 × 15 m “all-out” sprints (corresponding to a total of 625 m), with 20 s of passive recovery between efforts and 200 m of easy swimming between sets were included at the end of their swimming program over a 4 weeks period. Hypoxic condition was generated using a simulator pumping air with lowered oxygen concentration into a facial mask. An incremental maximal test on an ergocycle, as well as 100 m and 400 m freestyle swimming performance (real competition format) were assessed before (pre), 7 days (post-1), and 2 weeks (post-2) after intervention. During training, heart rate (HR) and oxygen saturation (SpO_2_) were monitored. RSH showed significantly lower SpO_2_ (70.1 ± 4.8% vs. 96.1 ± 2.7%, *P* < 0.01), concomitant with higher mean HR (159 ± 11 bmp vs. 141 ± 6 bmp, *P* < 0.01) than RSN. No significant changes in maximal oxygen uptake, other submaximal physiological parameters, 100 or 400 m swimming performances were found. Although providing additional physiological stress, performing in-water RSH does not provide evidence for higher benefits than RSN to improve swimmers performance.

## Introduction

Live low-train high (LLTH) is now a popular hypoxic training approach, for the simple reason that it does not disturb athletes' usual training routine (Girard et al., 2020). Few studies have investigated the effects of such LLTH methods on swimmers performance. To the best of the authors knowledge, dryland-based intermittent hypoxic training (IHT) (Czuba et al., 2017; Park et al., 2018) and in-water swimming-specific training using voluntary hypoventilation at low-lung volume (VHL) (Woorons et al., 2016; Trincat et al., 2017) or prolonged expiration and reduced-frequency breathing (Toubekis et al., 2017) have been reported. Research on IHT effects on swimmers' performance is not conclusive. On the one hand, 18 IHT sessions at a simulated altitude of 3,000 m enhanced VO_2max_ and 400 m freestyle performance in moderately trained swimmers (Park et al., 2018). On the other hand, 8 IHT sessions at a simulated altitude of 2,500 m were sufficient to enhance anaerobic capacity and swimming performance, despite no effect on VO_2max_ (Czuba et al., 2017). In this context, the hypoxic dose, type and training intensity, in addition to participants' training status could explain such controversial findings (McLean et al., 2014). Selection of training intensity was identified as a key factor for subsequent normoxic performance outcomes (McLean et al., 2014).

Consequently, another LLTH method, named repeated-sprint training in hypoxia (RSH), has been suggested as an effective alternative to improve aerobic and anaerobic performance in different disciplines. This method consists of the repetition of 3–4 sets of short “all-out” maximal-intensity exercise intervals (4–7 × 4–15 s), interspersed with passive recoveries (<30 s, exercise:recovery ratio 1:2–1:5) under hypoxic condition (2,900–3,500 m) for 2–5 weeks (2–3 sessions per week) (Brocherie et al., 2017; Millet et al., 2019). Under certain conditions, the RSH model could modify various aerobic-related exercise performance factors and lead to greater improvement compared with similar normoxic training (Brocherie et al., 2017). RSH training can increase mRNA expression of factors involved in pH regulation, glycolysis as well as in mitochondrial biogenesis (Faiss et al., 2013; Brocherie et al., 2017).

RSH requires sport-specific adjustment and exercise mode selection, because it may impact the magnitude of sport-specific fitness improvements, RSH would require some sport-specific adjustments and exercise mode selection. Using in-water swimming-specific exercise, Woorons et al. (2016) reported that 10 sessions of 12–20 × 25 m freestyle sprint with 15 s of recovery at a pace of 200 m freestyle permitted to improve 100, 200, and 400 m freestyle performance, mainly due to an in increase in the anaerobic glycolysis. Similarly, by making the VHL method more specific to swimming, with 2 sets of 16 × 15 m “all-out,” Trincat et al. (2017) demonstrated that the number of repeated sprints performed improved but without providing information about swimming performance. Although VHL led to strong desaturation (~88%), the “hypoxic dose” remain lower than when using systemic hypoxia (Woorons et al., 2016).

In this view, this study aimed to investigate the effect of a 4 weeks in-water swimming-specific repeated-sprint in hypoxia (RSH) compared to similar training in normoxia (RSN).

## Materials and Methods

### Participants

A total of 10 trained swimmers were recruited (16.67 ± 1.97 years, 1.61 ± 6.44 cm, 56.34 ± 7.38 kg, 22.74 ± 3.10 kg/m^2^). All swimmers were members of a swimming club and were tested according to their annual periodization plan. The subjects followed their usual training programmes during the study. Swimmers' level was classified according to De Pauw et al. (2013). All participants were informed about the equipment and experimental design. Consent forms were signed by all participants and parents/guardians for minors before initiating the study. Procedures performed were in accordance with the 1964 Helsinki declaration and its later amendments or comparable ethical standards. The Bioethical and Biosecurity Commission of the University of Extremadura approved this study protocol (registration number: 13/2016).

### Experimental Design

Following a repeated-measures, counterbalanced cross-over design, participants were requested to perform two trials consisting of in-water repeated sprints in hypoxic [RSH, 4,040 m (FiO_2_ = 13.7%)] or normoxic [RSN, 459 m (FiO_2_ calibrated and reset = 20.9%)] condition.

After familiarization that include anthropometric measurements and testing and training protocols trials, participants were assessed before (pre), 7 days (post-1), and 2 weeks (post-2) after the intervention by researchers blind to treatment assigned.

### Repeated-Sprint Intervention

After pre-tests, participants were requested to add 8 RSH or RSN training, 2 times per week with at least 48 h in-between over a 4 weeks period. Exercise consisted of 3 sets of 5 × 15 m “all-out” sprints (for a total of 625 m) with 20 s of passive recovery between efforts and 200 m of easy swimming between sets and was performed at the end of a swimming session (volume of ~5,000 m swam). For RSN, no material was used or connected to blind the participants who were performing at 459 m where FiO_2_ measurement was calibrated and reset to 20.9%. For RSH, normaboric hypoxia [fraction of inspired oxygen (FiO_2_) 13.7 ± 0.3%] was produced by a hypoxic generator with a semi-permeable filtration membrane (nitrogen filter technique; CAT 310, Louisville, Colorado, USA) connected to a waterproof facial mask. In both conditions, FiO_2_ was controlled using an electronic device (HANDI+, Maxtec, Salt Lake City, Utah, USA). Simulated altitude for RSH (4,040 m) was calculated according to chart and guidelines provided by hypoxic generator manufacturer, based on FiO_2_ (13.7%) and considering that for a reset value of FiO_2_ = 20.9 %, altitude was 459 m instead of sea level.

During each RSH/RSN session, adherence, exercise workloads and physiological responses were recorded on a daily training log. After each set, SpO_2_ was controlled using a pulse-oximeter (Konica Minolta, Japan). HR was also continuously monitored (OH1+, Polar, Finland). Participants were also asked to report their rated perceived exertion (RPE; 0–10 value) score at the end of each training session.

### Testing Protocol

At the 3 designated time points, lab testing and anthropometrics (body weight and height to the nearest 0.1 kg and 0.5 cm, respectively) were assessed. Before attaching the facial mask for gas analysis (Metalyzer 3b, CORTEX Biophysik GmbH, Leipzig, Germany), a 5-min ergocycling warm-up at 50 W and 1 min of rest. Participants started cycling at 60 W and the work rate was increased by 30 W every 3 min until exhaustion. The flow sensor and the gas analysers were calibrated using a 3-L syringe and calibration gas (O_2_ 16.10 and 20.93%; CO_2_ 0.00 and 5.20%) before each test. Fluctuations of breath-by-breath data were minimized using 6 breaths smoothing and consequent 30 s averaging, as recommended by the manufacturer. Respired gas was sampled continuously from the mouthpiece and analyzed for fractional concentrations of O_2_ and CO_2_. HR was recorded continuously during the test using an HR monitor (Polar H7 HR, Polar Electro Oy, Kempele, Finland).

Oxygen uptake (VO_2_) was considered as maximal (VO_2max_) when at least three of the following four criteria were met: (1) a plateauing of VO_2_ (defined as an increase of no more than 2 mL·kg^−1^·min^−1^ with an increase in workload) during the later stages of the exercise test; (2) a HR>90% of the predicted maximum for their age (220 – age); (3) a respiratory exchange ratio (RER) > 1.1; and (4) an inability to maintain the minimal required pedaling frequency (i.e., 60 rpm) despite maximum effort and verbal encouragement. The figures for absolute VO_2_, absolute workload, time to exhaustion (TtE), maximal heart rate (HR_max_), and RER and were obtained. VO_2max_ was calculated as the average oxygen uptake over the last 60 s of the test. Peak power was defined as the maximal power achieved in the course of the last 3 min step completed during the incremental test. Anaerobic threshold and aerobic threshold were calculated based on the aforementioned results. Metabolic thresholds were determined manually via ventilatory gases using the following criteria: the first rise in the ventilatory equivalent for O_2_ without concomitant rise of ventilatory equivalent for CO_2_ for aerobic threshold, and V-slope method for anaerobic threshold. In the last 3 min of recovery, blood from arterialized capillary blood (finger) was taken to determine lactate levels using lactate analyser (Lactate Scout, SensLab GmbH, Germany).

Freestyle 100 and 400 m swimming performance was evaluated at pre, post-1, and post-2 in an official competition 48 h prior to laboratory testing. Omega Electronics OCP5 touchpad (Corgémont, Switzerland) was used to measured official swimming performance, which was extracted from race time reports.

### Statistical Analysis

Statistical analyses were performed using statistical package SPSS v.20 for MAC (IBM, New York, USA). Data are presented as mean ± SD. Kolmogorov–Smirnov tests were realized to show distribution of the studied variables and Levene's test for homogeneity of variance. Two-way analysis of variance (ANOVA) with repeated measures [Condition (RSH vs RSN) × Time (Pre- vs. Post-1 vs. Post-2)] was used to compare variables. When ANOVA revealed a significant interaction or main effect, Bonferroni *post-hoc* analysis was used to identify where changes occurred. In all analyses, *P* < 0.05 was taken to indicate significance. The effect size (d Cohen) was calculated for all variables. The magnitude of the difference was considered as a small (0.2), moderate (0.5), or large (0.8) effect size (Cohen, 1992).

## Results

**Figure 1:**
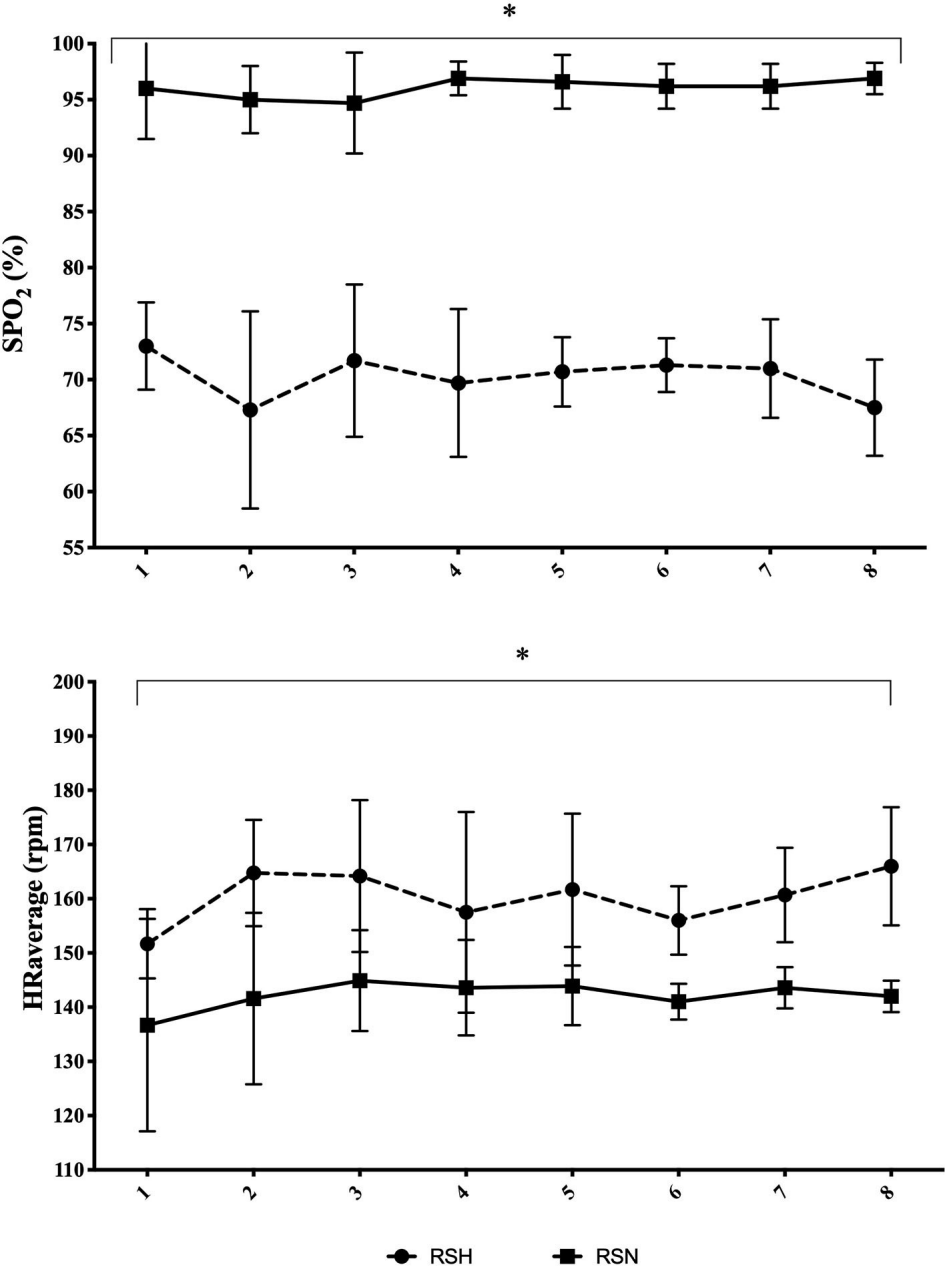
Time courses of changes in SpO_2_ and mean HR over the RSH/RSN intervention. Mean ± standard deviation. *P < 0.01 vs. RSN.

**Figure 2:**
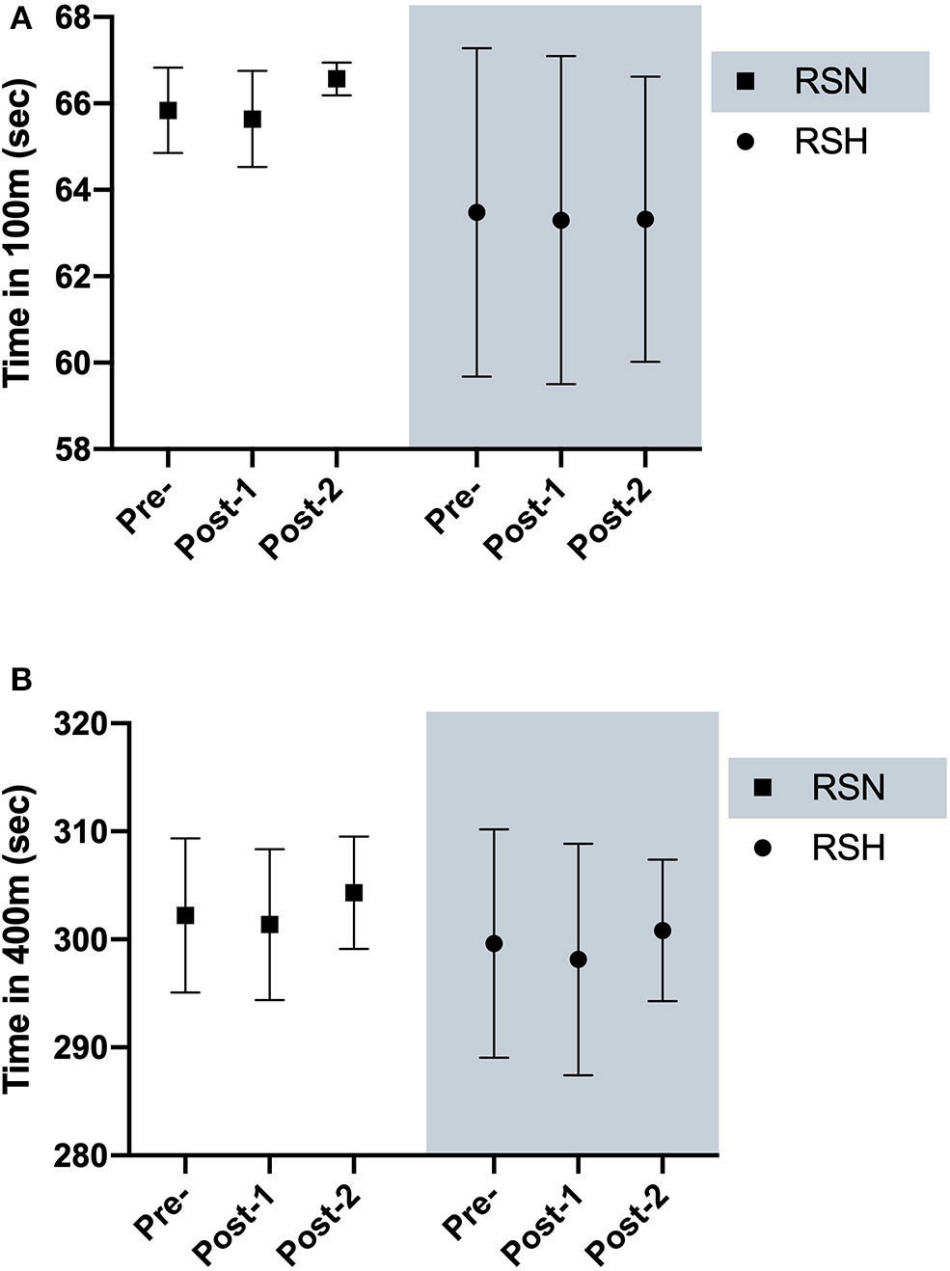
Swimming 100 m **(A)** and 400 m **(B)** freestyle performance. Mean ± standard deviation before (pre), 7 days (post-1), and 2-weeks (post-2) after intervention.

There was a significant interaction (condition x time) in both SpO_2_ and mean HR over the eight training sessions of training (Figure 1). RSH showed significant lower values of SpO_2_ and higher mean HR in all sessions (*P* < 0.01) compared with RSN. No significant changes were found for 100 and 400 m freestyle swimming performance at pre, post-1, and post-2 (Figure 2).

No significant interaction (condition x time) was found for Lactate, VO_2max_, absolute workload, TtE, HR_max_, and VE at baseline, post-training or detraining (Table 1). Within-group analysis reveals non-significant decrease in HR_max_ (*P* = 0.061, *d* = 1.42) and in VO_2max_ (*P* = 0.071, *d* = 0.67) for RSH. For RSN, HR_max_ (*P* < 0.001, *d* = 2.36) and VO_2max_ (*P* < 0.01, *d* = 1.50) significantly decreased at post-1. This was accompanied by significant increase (*p* < 0.001) in lactate with larger effect size (*d* = 1.55) between pre and post-2.

**Table 1:** Exercise testing maximal parameters before (pre), 7 days (post-1), and 2-weeks (post-2) after intervention.

		**Pre**	**Post-1**	**% Δ**	**d Cohen**	**Post-2**	**% Δ**	**d Cohen**	**Mixed factorial ANOVA**, ***p*****-values**		
				**(Pre-Post 1)**			**(Pre-Post 2)**		**Time**	**Condition**	**Time × condition**
Lactate, Mmol	RSH	9.0 ± 1.6	10.6 ± 4.0	17.8	0.57	11.9 ± 6.7	32.2	0.24	0.055	0.676	0.417
	RSN	9.2 ± 1.5	9.7 ± 2.2	5.4	0.27	14.5 ± 4.0^*^	57.6	1.55	0.007		
VO_2max_, L·min^−1^	RSH	2.9 ± 0.2	2.7 ± 0.4	−6.9	0.67	2.6 ± 0.3	−10.3	0.29	0.071	0.475	0.081
	RSN	2.8 ± 0.2	2.5 ± 0.2^**^	−10.7	1.50	2.6 ± 0.2	−7.1	0.50	0.012		
Absolute workload, watts	RSH	198.0 ± 16.4	192.0 ± 16.4	−3.0	0.37	192.0 ± 16.4	−3.0	0.00	0.375	0.421	0.709
	RSN	190.0 ± 15.5	180.0 ± 26.8	−5.3	0.47	190.0 ± 15.5	0.0	0.47	0.432		
TtE, min	RSH	15.6 ± 1.8	15.6 ± 2.0	0.0	0.00	15.2 ± 1.2	−2.6	0.25	0.844	0.652	0.553
	RSN	15.1 ± 1.8	14.6 ± 1.8	−3.3	0.28	15.4 ± 1.9	2.0	0.43	0.822		
HR_max_, beats·min^−1^	RSH	192.5 ± 3.1	182.7 ± 10.7	−5.1	1.42	183.7 ± 9.3	−4.6	0.10	0.068	0.754	0.735
	RSN	193.5 ± 4.0	183.0 ± 4.9^***^	−5.4	2.36	186.3 ± 10.0	−3.7	0.44	0.021		
RER	RSH	1.19 ± 0.06	1.21 ± 0.08	1.7	0.29	1.20 ± 0.04	0.8	0.17	0.403	0.997	0.223
	RSN	1.20 ± 0.05	1.17 ± 0.06	−2.5	0.55	1.23 ± 0.06	2.5	1.00	0.354		

*Mean ± standard deviation; VO_2max_ (maximal oxygen uptake), TtE (time to exhaustion), HR_max_ (maximal heart rate), RER (respiratory exchange ratio). RSH, repeated-sprint training in hypoxia; RSN, repeated-sprint training in normoxia*. ^*^Indicates differences with respect to baseline (post-hoc t-test with Bonferroni correction):

* P < 0.05,

** P < 0.01,

*** *P < 0.001*.

No significant interaction was found at the anaerobic threshold for absolute VO_2_, absolute workload, time and HR_max_ (Table 2). At aerobic threshold, both RSH and RSN showed a significantly lower VO_2max_ at post-1 and post-2 in reference to pre, with large and moderate effect size for RSH and RSN, respectively (Table 3).

**Table 2:** Exercise testing anaerobic threshold parameters before (pre), 7 days (post-1), and 2-weeks (post-2) after intervention.

		**Pre**	**Post-1**	**% Δ**	**d Cohen**	**Post-2**	**% Δ**	**d Cohen**	**Mixed factorial ANOVA**, ***p*****-values**		
				**(Pre-Post 1)**			**(Pre-Post 2)**		**Time**	**Condition**	**Time × condition**
VO_2_, L·min^−1^	RSH	2.3 ± 0.2	2.2 ± 0.3	−4.3	0.40	2.1 ± 0.2	−8.7	0.40	0.211	0.553	0.679
	RSN	2.2 ± 0.2	2.0 ± 0.3	−9.1	0.80	2.1 ± 0.3	−4.5	0.33	0.225		
Absolute Workload, watts	RSH	168.0 ± 16.4	168.0 ± 16.4	0.0	0.00	168.0 ± 16.4	0.0	0.00	0.906	0.517	0.903
	RSN	160.0 ± 15.5	160.0 ± 24.5	0.0	0.00	165.0 ± 25.1	3.1	0.20	0.903		
Time, min	RSH	12.3 ± 1.6	12.3 ± 1.6	0.0	0.00	12.30 ± 1.6	0.0	0.00	0.905	0.518	0.906
	RSN	11.5 ± 1.5	11.5 ± 2.4	0.0	0.00	12.0 ± 2.5	4.3	0.20	0.903		
HR_max_, beats·min^−1^	RSH	174.2 ± 8.8	173.5 ± 10.6	−0.4	0.07	171.7 ± 12.2	−1.4	0.16	0.656	0.790	0.816
	RSN	173.7 ± 6.9	170.7 ± 5.6	−1.7	0.48	171.3 ± 9.8	−1.4	0.08	0.691		

*Mean ± standard deviation; VO_2_ (absolute oxygen uptake), HR_max_ (maximal heart rate). RSH, repeated-sprint training in hypoxia; RSN, repeated-sprint training in normoxia*.

**Table 3:** Exercise testing aerobic threshold parameters before (pre), 7 days (post-1), and 2-weeks (post-2) after intervention.

		**Pre**	**Post-1**	**% Δ**	**d Cohen**	**Post-2**	**% Δ**	**d Cohen**	**Mixed factorial ANOVA**, ***p*****-values**		
				**(Pre-Post 1)**			**(Pre-Post 2)**		**Time**	**Condition**	**Time × condition**
VO_2_, L·min^−1^	RSH	1.7 ± 0.1	1.6 ± 0.1^*^	−5.9	1.00	1.6 ± 0.1^*^	−5.9	0.00	0.023	0.485	0.539
	RSN	1.7 ± 0.2	1.6 ± 0.1^*^	−5.9	0.67	1.6 ± 0.1^*^	−5.9	0.00	0.025		
Absolute Workload, watts	RSH	120.0 ± 0.0	120.0 ± 0.0	0.0	0.00	120.0 ± 0.0	0.0	0.00	0.389	0.389	0.401
	RSN	115.0 ± 12.2	115.0 ± 12.2	0.0	0.00	120.0 ± 0.0	4.3	0.82	0.401		
Time, min	RSH	7.5 ± 0.0	7.5 ± 0.0	0.0	0.00	7.5 ± 0.0	0.0	0.00	0.388	0.388	0.389
	RSN	7.0 ± 1.2	7.0 ± 1.2	0.0	0.00	7.5 ± 0.0	7.1	0.83	0.402		
HR_max_, beats·min^−1^	RSH	150.2 ± 5.9	150.0 ± 8.0	−0.1	0.03	148.5 ± 8.3	−1.1	0.18	0.887	0.594	0.790
	RSN	148.2 ± 8.0	146.7 ± 6.4	−1.0	0.21	148.2 ± 4.7	0.0	0.27	0.918		

Mean ± standard deviation; VO_2_ (absolute oxygen uptake), HR_max_ (maximal heart rate). RSH, repeated-sprint training in hypoxia; RSN, repeated-sprint training in normoxia. ^*^Indicates differences with respect to baseline (post-hoc t-test with Bonferroni correction):

* *P < 0.05*.

## Discussion

To the best of authors knowledge, this is the first study investigating in-water swimming-specific RSH using systemic normobaric hypoxia. Findings indicate that a 4 weeks RSH period (i.e., 8 additional sessions, 3 sets of 5 × 15 m “all-out” sprint, 20 s active recovery between sprint, 200 m of easy swimming between sets performed at a 4,040 m, FiO_2_ 13.7 ± 0.3%) did not elicit higher 100 and 400 m freestyle swimming performance, despite higher physiological stress (i.e., mean HR and SpO_2_) during training compared with RSN.

Normobaric hypoxia resulted in larger hypoxemia (i.e., lower SpO_2_ in RSH vs. RSN) during RSH, which would increase the demand on anaerobic pathway to maintain ATP provision rate (Girard et al., 2017). In this view, exposing swimmers to normobaric hypoxia through hypoxic generator connected via a facial mask appears effective and offer alternative for aquatic sports training. However, it is noteworthy that such procedure may have somewhat altered swimming capability due to movement restriction from the system. Added to the large physiological stress [i.e., mean HR elevation and extreme desaturation (67–74% SpO_2_)] imposed by the hypoxic level selected (4,040 m, FiO_2_ 13.7 ± 0.3%), this may have resulted in unexpected outcomes than those generally reported in RSH studies using altitude ranging 2,200–3,500 m (Brocherie et al., 2017; Millet et al., 2019). It may indicate that RSH training at too high altitude (>4,000 m) might compromise swimming and add negative outcomes (Goods et al., 2014).

Using lower hypoxic stress with RSH-VHL method, Trincat et al. (2017) showed that SpO_2_ could decrease below 84% but return to normoxic values during recovery periods, resulting in a mean SpO_2_ of 94.5% without any impact on HR during a RSH-VHL set in competitive swimmers. Interestingly, they reported RSH-VHL-induced benefits in repeated-sprint ability after 6 RSH-VHL sessions. As such, this may indicate that the hypoxic stress imposed in the present study was too high, confirming that the more is not better (Goods et al., 2014). While there is no guarantee that repeated-sprint ability transfer in freestyle performance in swimmers, Woorons et al. (2016) demonstrated that similar RSH-VHL protocol (i.e., 12–20 × 25 m at supramaximal intensity) improved 100, 200, and 400 m freestyle performance in triathletes.

It has been reported that RSH generates peripheral adaptations and may further improve performance compared with RSN (Brocherie et al., 2017; Girard et al., 2017). As previously mentioned, despite RSH-induced acute physiological responses, participants' swimming performance was not improved, neither after intervention (post-1), nor after 2 weeks (post-2). Swimming 100 and 400 m freestyle mainly rely on lactate production and tolerance, and glycolytic metabolism and VO_2max_, respectively. Accordingly, RSH-induced molecular adaptation should transfer in higher peripheral glycolytic activity (Faiss et al., 2013). Unfortunately, lactate level could not be measured during 100 and 400 m freestyle trials to confirm (or not) a change in anaerobic metabolism. That said, lactate measured after the maximal incremental test did not differ between RSH and RSN. Neither maximal nor submaximal variables in aerobic and anaerobic threshold were affected by RSH. Despite possible effect of RSH on aerobic performance (Brocherie et al., 2017), it seems that maximal or high-intensity exercise of duration shorter than 4 min is not sufficient to improve aerobic capacity, even if anaerobic performance could be enhanced (Roels et al., 2005). Inversely, moderate intensity and large volume (20–30 min) exercise may favor aerobic capacity improvement (Dufour et al., 2006; Zoll et al., 2006). Another point to mention is that the maximal incremental test was not sport-specific and really uncommon for our participants. With an increment of 30 W every 3 min (corresponding to approximately +15%), such protocol may not have been sufficiently sensitive for the participants and may explain the lack of differences found in the present study. As such, it seems advisable that performance testing and RSH training for swimmers should be performed in-water (Czuba et al., 2017) or at least using sport-specific principles.

Training level and background of participants are important variables to consider when programing LLTH high-intensity hypoxic-specific swimming protocols (Woorons et al., 2016). Further, in order to adequately stimulate adaptive changes, adjusted exercise intensity within a sufficient training volume is needed (Czuba et al., 2017). With water density increasing the energy demand per unit of distance (Pendergast et al., 2003), it is possible that the RSH protocol developed in the present study was inadequate and had led to understimuli (i.e., too much hypoxic stress altering exercise performance) or overstimuli (i.e., exaggerated fatigue development leading to overtraining). Interestingly, Brechbuhl et al. (2018) reported no effect of RSH (6 sessions during a 14-day shock microcycle, 4 sets of 5 × 6 s repeated-shuttle sprints with 24 s of passive recovery) 3 days after intervention in a rookie professional tennis player program. But 21 days later, both fitness and tennis performance gains were observed, interpreted by dissipation of temporary fatigue state (Brechbuhl et al., 2018). Further investigations are warranted to better delineate RSH protocol (e.g., hypoxic dose, number of sets, repetition, exercise:recovery ratio).

To conclude, in-water swimming-specific repeated-sprint training in normobaric hypoxia induced larger physiological stress compared with similar training performed in normoxia, thereby indicating its applicability in aquatic sports. However, such demanding protocol did not result in improved 100 and 400 m freestyle performance in young moderate-level swimmers, indicating the importance to carefully design and monitor training in reference to participants' profile and sport demand.

## Data Availability Statement

The datasets generated for this study are available on request to the corresponding author.

## Ethics Statement

The studies involving human participants were reviewed and approved by the University of Extremadura. Written informed consent to participate in this study was provided by the participants' legal guardian/next of kin.

## Author Contributions

GO: conceptualization. GO, MC-C, AC-C, and VZ: methodology and formal analysis. GO, VZ, and AG-C measurements and data field acquire. MC-C, AC-C, VZ, and AG-C: writing—original draft preparation and writing—review and editing. All authors contributed to the article and approved the submitted version.

## Conflict of Interest

The authors declare that the research was conducted in the absence of any commercial or financial relationships that could be construed as a potential conflict of interest.
